# Supplementary material for: Chromothripsis during telomere crisis is independent of NHEJ, and consistent with a replicative origin
Source: Genome Res. 2019 May;29(5):737–49. doi: 10.1101/gr.240705.118 (PMC6499312; doi:10.1101/gr.240705.118)
Supplement: Supplemental Material [file supp_gr.240705.118_Supplemental_file_1.zip › contigs/annotated_contigs/DB37/contig.3.DB37_length_401_mean_cov_5.99002493766.docx]

**DB37_length_401_mean_cov_5.99002493766**

```
GGGGGGGGGGGGGGGGGTGGGGGGGGGGGGGGGGGGGGGGGGGGGGGGGGGGGGGGGGGGGCTGGGGCG|CGGGGTGGGGTG|GGGCGC
 >chr2:33141586-33141655 + E=5e-21 p=0e+00                                        >chr1
AGTGGGGAGGTTGGGCGCAGGGCGGGGTTGGGCGCTGGCGGGGTGCTTGAGCGCTGAGGCTTTAGCTCCATGGGAGCAGGAGGTAATGG
0:1671492-1671703 - E=1e-115
GGGAAGTGAATTAGAATGTCACTGTGAAGTCCTGACCATCACGATGAGGAGGTCTCCACCAATCCCAGGGGACAGCATGGCTAGGAGTA

AACCCACACTTGTCTCAGAAACTGATT|AG|AATGTTTAGAATATATCTTTAAGCTTCATATCTAAAGGAGAGACCTATGTCTATTTAA
                              >chr10:17627730-17627837 + E=7e-53
ATATTTTGTAAATTTAGACTAAAACTATAAACAAATTGAATTTTAAGTT
```
